# Redox Status and Muscle Pathology in Rheumatoid Arthritis: Insights from Various Rat Hindlimb Muscles

**DOI:** 10.1155/2019/2484678

**Published:** 2019-03-26

**Authors:** A. B. Oyenihi, T. Ollewagen, K. H. Myburgh, Y. S. L. Powrie, C. Smith

**Affiliations:** Department of Physiological Sciences, Stellenbosch University, South Africa

## Abstract

Due to atrophy, muscle weakness is a common occurrence in rheumatoid arthritis (RA). The majority of human studies are conducted on the *vastus lateralis* muscle—a muscle with mixed fiber type—but little comparative data between multiple muscles in either rodent or human models are available. The current study therefore assessed both muscle ultrastructure and selected redox indicators across various muscles in a model of collagen-induced rheumatoid arthritis in female Sprague-Dawley rats. Only three muscles, the *gastrocnemius*, *extensor digitorum longus* (*EDL*), and *soleus*, had lower muscle mass (38%, 27%, and 25% loss of muscle mass, respectively; all at least *P* < 0.01), while the *vastus lateralis* muscle mass was increased by 35% (*P* < 0.01) in RA animals when compared to non-RA controls. However, all four muscles exhibited signs of deterioration indicative of rheumatoid cachexia. Cross-sectional area was similarly reduced in *gastrocnemius*, *EDL*, and *soleus* (60%, 58%, and 64%, respectively; all *P* < 0.001), but *vastus lateralis* (22% smaller, *P* < 0.05) was less affected, while collagen deposition was significantly increased in muscles. This pathology was associated with significant increases in tissue levels of reactive oxygen species (ROS) in all muscles except the *vastus lateralis*, while only the *gastrocnemius* had significantly increased levels of lipid peroxidation (TBARS) and antioxidant activity (FRAP). Current data illustrates the differential responses of different skeletal muscles of the hindlimb to a chronic inflammatory challenge both in terms of redox changes and resistance to cachexia.

## 1. Introduction

There can be no doubt that individuals suffering from rheumatoid arthritis (RA) have significantly decreased quality of life. In addition to the chronic pain and other primary symptoms arising from the inflammatory processes in joints, the majority of patients also report skeletal muscle weakness [1]. However, there is a disconnection between the degree of rheumatoid cachexia (defined as arthritis-associated loss of muscle mass with little or no loss of fat mass [2]) and the relatively more severe degree of muscle weakness experienced. Initially, the more severe loss of strength was ascribed to joint deformation and pain [3], but more recently, contractile dysfunction, mediated by tumor necrosis factor (TNF), was implicated, with TNF reported to decrease specific force by increasing cytosolic oxidant activity in the muscle [4].

The pathology of skeletal muscle in patients with RA is clearly complex and much research has already been conducted in this context, so that at least a partial picture of role players is available. For example, while neuromuscular fatigue (assessed by electromyography) was reported to weakly correlate with subjective perception of fatigue and physical activity level, it did not correlate to either clinical profile or treatment features [5]. This suggests that while advancement of emotional well-being should form part of treatment strategy, neuromuscular pathology is probably not a major role player in RA. In contrast, increased muscle inflammatory cytokine levels, altered expression of genes involved in muscle repair and glycolytic metabolism, as well as increased levels of fibrosis-associated amino acids, correlated with disease progression, physical inactivity, and pain in a large cohort of RA patients [6]. These data, generated from the *vastus lateralis* muscle, and specifically the finding related to altered glycolytic metabolism, raised the question of whether different muscles or muscles with different fiber type distributions may be differentially sensitive to RA-associated pathology.

Given both the significant implication of inflammation in RA and the interlinked nature of inflammation and oxidative stress, attention should also be extended to redox status, in order to form a more holistic picture of pathological maladaptations that could potentially be targeted by intervention. In an elegant study on the slow-twitch *soleus* muscle from rats subjected to collagen-induced arthritis (CIA), peroxynitrite-induced oxidative damage to myofibrillar proteins was implicated in measured deficits of muscle force production, such as shorter maximal contraction velocity and slower twitch contraction and relaxation [7]. The same group subsequently illustrated similar deficits in force production—also ascribed to reactive nitrogen species—in the fast-twitch *extensor digitorum longus* (*EDL*) muscle [8]. These studies sketch a fairly fiber-type independent picture of muscle pathology in this model. However, these observations were made at a relatively advanced time point (≈day 45, with last booster shot on day 28) and do not allow for direct interpretations on specific mechanisms at play during the earlier, disease development phase. Furthermore, the degree to which different muscle types are compromised in CIA has not been comprehensively and comparatively assessed.

The aim of the current study was therefore to assess muscle morphology and selected aspects of muscle pathology and redox changes in four different hindlimb muscles in rats with collagen-induced arthritis. A somewhat milder model (day 35, with last booster shot on day 8) was employed in order to elucidate the extent to which free radical involvement and endogenous antioxidant mechanisms may contribute to early pathology in different muscles.

## 2. Materials and Methods

### 2.1. Ethics Statement and Animal Handling

The Stellenbosch University Animal Research Ethics Committee ethically cleared this study (Protocol number: SU-ACUD17-00034). Twenty (20) female Sprague-Dawley rats weighing 180–200 g were procured from the Stellenbosch University small laboratory animal breeding facility. They were housed in groups of 5 rats per cage in a temperature- and humidity-controlled room (23 ± 1°C, 40–60% humidity) with a set 12 h light-dark cycle and fed standard commercially available rat chow and tap water *ad libitum*. After acclimatization to the new environment for about 7 days, rats were randomly divided into two groups of 10 rats each—normal control (NC) and collagen-induced rheumatoid arthritis (RA). All experimental animals received humane care according to the principles outlined in the National Research Foundation *Guide for the Care and Use of Laboratory Animals*.

### 2.2. Collagen-Induced Rheumatoid Arthritis Model

#### 2.2.1. Chemicals

Bovine collagen type II, incomplete Freund's adjuvant, and rat anti-collagen IgG ELISA kit were purchased from Chondrex Inc., WA, USA. Isoflurane (Isofor) was purchased from Safeline Pharmaceuticals, Johannesburg, South Africa. All other chemicals and reagents used in this study were of analytical grade and purchased from Sigma-Aldrich (MO, USA) or Merck (Darmstadt, Germany) unless otherwise stated.

#### 2.2.2. Experimental Design

The well-established rat collagen-induced arthritis (CIA) method [9, 10] was used to induce arthritis in the RA group. Briefly, bovine heterologous type II collagen was first dissolved in 0.01 N glacial acetic acid (2 mg/ml) before an emulsion was prepared using an equal volume of incomplete Freund's adjuvant. This emulsion was slowly injected intradermally twice just above the tail region of each rat under isoflurane anesthesia, 7 days apart. The time of onset of swelling in rat paws was recorded. The progression of clinical symptoms was monitored daily and scored as follows: 0 = no swelling; 1 = erythema and digits swollen; 2 = erythema, digits, and pad swollen; and 3 = erythema, also digits, pad,joints and entire leg swollen. The total score for each rat was given as the addition of all affected paws, so the highest attainable score was 10 for each rat, as previously described [11].

After the 5-week experimental period, all rats were killed by guillotine decapitation and the trunk blood was immediately collected into tubes via a heparinized funnel. The plasma was subsequently separated after centrifugation at 2,000g for 10 minutes using a Spectrafuge 24D centrifuge (Labnet International Inc., NJ, USA). The *EDL*, *gastrocnemius*, and *soleus* and *vastus lateralis* muscles from the hindquarters of each rat were removed carefully, weighed, snap-frozen in liquid nitrogen, and then stored at -80°C until subsequent analysis.

#### 2.2.3. Validation of Model via Plasma Anti-Collagen IgG Titer

The successful induction of arthritis in rats was confirmed by the production of antibodies to type II collagen using the rat anti-collagen IgG ELISA kit (Chondrex Inc., WA, USA) and following the manufacturer's protocol.

### 2.3. Muscle Histology

Frozen tissues (*EDL*, *gastrocnemius*, and *vastus lateralis*) were sectioned in 10 *μ*m cross sections with a cryostat (Leica CM1860 UV, Leica Biosystems Nussloch GmbH, Germany) at -25°C and stored at -20°C. To ensure consistency between samples, a predetermined section was cut off the proximal end of each sample (differed between muscle types) before sectioning occurred. This is particularly important in the *vastus lateralis* as different fiber type proportions exist in the different areas (superficial vs. deep and proximal vs. distal) [12]. Sections were allowed to thaw at room temperature for 20 minutes before staining.

#### 2.3.1. Hematoxylin and Eosin Staining

H&E staining was used to view the overall muscle structure. Hematoxylin binds to and stains all DNA/RNA structures blue. Eosin counterstains all proteins of the tissue pink. Slides were submerged in the following order for one minute each, excluding eosin which was stained for 30 seconds: dH_2_O, two changes of Mayer's hematoxylin, warm tap water, Scott's tap water, dH_2_O, eosin, H_2_O, 95% ethanol, 100% ethanol, and finally clearance in xylene. Once stained, the slides were mounted with mounting media (DPX, 06522, Sigma-Aldrich, USA) and covered with a cover slip for viewing.

#### 2.3.2. Picrosirius Red Staining

Sirius red is a polyazo dye which is specifically used for staining collagen. The stain dyes collagen bright red, leaving muscle fibers, cytoplasm, and red blood cells a lighter yellow/red color. Picrosirius red differs from sirius red staining with the addition of picric acid which prevents the indiscriminate staining of noncollagenous structures by sirius red.

Sections were fixed in neutral buffered formalin for 30 minutes. Slides were rinsed in dH_2_O and stained with Weigert's hematoxylin for 8 minutes. Sections were washed in 3 changes of water followed by staining with picrosirius solution for one hour. Picrosirius solution was made up of 0.5 g sirius red F3B (CI35780, Sigma-Aldrich, USA) in 500 ml saturated aqueous picric acid (197378, Sigma-Aldrich, USA). Sections were washed in 0.5% acetic acid (A6283, Sigma-Aldrich, USA) in dH_2_O twice for 5 minutes each. Sections were then dehydrated in changes of ethanol (70%, 95%, and 100%) and then cleared in xylene (296325, Sigma-Aldrich, USA), and cover slips were mounted with mounting media (DPX, 06522, Sigma-Aldrich, USA).

#### 2.3.3. Image Acquisition

All histological slides were viewed using bright-field microscopy (Nikon Eclipse E400), mounted with a camera (Nikon DS-Fi2), and processed through a digital sight processor (DS-U3, Nikon, Japan). Image processing was done on Nikon Instruments Software (NIS-Elements v4.10) on a desktop computer (Dell, USA) running Windows 7 (Microsoft, USA). Images were taken at 100x and 200x magnifications (magnification was calculated from ocular lens (10x) multiplied by objective lens (10x/20x)).

#### 2.3.4. Image Analysis

The cross-sectional area of the fibers was measured using H&E sections and ImageJ software (version 1.49, Wayne Rasband). 50 fibers per sample were measured. In order to assess fibrosis, picrosirius red images were analyzed using ImageJ software (version 1.49, Wayne Rasband) with the color deconvolution plug-in as developed by Landini (version 1.5). The picrosirius stains were processed using the ImageJ RGB option. Briefly, the plug-in unmixes the RGB image into three 8-bit images with a color lookup table that corresponds to the respective vector colors. The analysis measurements were set to measure “area,” “area fraction,” “limit to threshold,” and “display label.” The threshold of each of the three images was adjusted allowing the measurement of (1) the background, (2) the connective tissue, and (3) total tissue. Percentage of fibrosis in the tissue (×100 magnification) was calculated as follows: (1) background was subtracted from both the connective tissue and total tissue and (2) the following formula was used: percentage of fibrosis = (connective tissue/(connective tissue + total tissue))∗100.

### 2.4. Sample Analysis for Redox Status

#### 2.4.1. Oxidative Stress

Frozen *vastus lateralis*, *soleus*, *EDL*, and *gastrocnemius* tissues were thawed on ice and homogenized 100 mg/ml in 10 mM phosphate-buffered saline (PBS, pH 7.2) and centrifuged at 10,000g for 15 min at 4°C to obtain the supernatants used for the analyses. The presence of reactive oxygen species (ROS) in tissue homogenates was evaluated by the ROS-dependent oxidation of the nonfluorescent 2′,7′-dichlorofluorescein- (DCF-) DiOxyQ probe to the highly fluorescent DCF using the OxiSelect™ ROS assay kit (Cell Biolabs Inc., CA, USA) and following the manufacturer procedure. The ferric ion reducing antioxidant power (FRAP) value in all muscle homogenates was determined by measuring the reduction of ferric tripyridyltriazine (Fe^3+^-TPTZ) complex to the ferrous (Fe^2+^) form by antioxidants as detailed earlier by Benzie and Strain [13]. This is monitored by the change in absorption at 593 nm in a SPECTROstar Nano® absorbance plate reader (BMG Labtech, Ortenberg, Germany). Lipid peroxidation in muscle homogenates was evaluated by the formation of the stable product—malondialdehyde (MDA)—in a reaction medium containing thiobarbituric acid (TBA). We used the method described by Varshney and Kale [14] but with slight modifications: briefly, the MDA formed in tissues highly reacts with TBA under acidic conditions to form a complex that is better purified by the addition of butanol and saturated sodium chloride and absorbs maximally at 532 nm using a SPECTROstar Nano® absorbance plate reader (BMG Labtech, Ortenberg, Germany).

### 2.5. Statistical Analysis

Effects were compared for statistical significance using Student's *t*-tests or one- or two-way analysis of variance (ANOVA) as appropriate, with Bonferroni post hoc tests where applicable. Data are presented as means and standard deviations, unless otherwise indicated. Statistical significance was set at *P* < 0.05.

## 3. Results

**Figure 1:**
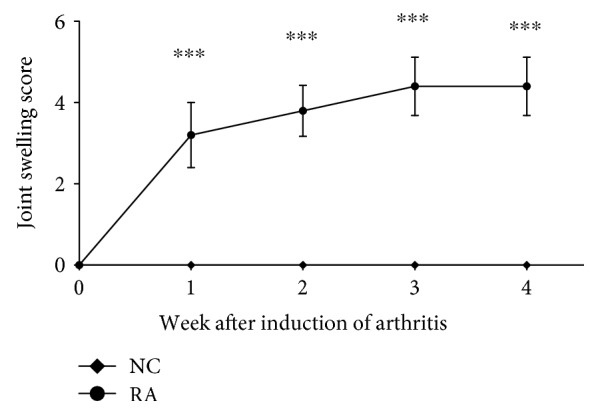
Weekly scores indicative of clinical symptoms of arthritis development in a collagen-induced rheumatoid arthritis model in female Sprague-Dawley rats (*n* = 10 per group). Statistical analysis: one-way ANOVA with repeated measures and Bonferroni post hoc test. ^∗∗∗^, *p* < 0.0001 significantly different from controls.

Successful induction of relatively mild rheumatoid arthritis was confirmed by clinical observations (Figure 1). Onset of joint swelling was typically within 11-18 days after the initial exposure to collagen. In addition, significantly elevated levels of anti-collagen antibody titer were measured in RA animals (0.02 ± 0.007 g/ml plasma; *P* < 0.0001) when compared to non-RA controls, in which antibody levels were not detectable.

**Figure 2:**
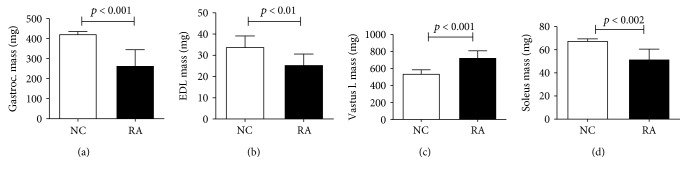
Muscle mass of different rat hindlimb skeletal muscles on day 35 of CIA (*n* = 10 per group). Bars indicate mean mass of muscle (left and right muscle weights were averaged for each animal) and error bars are standard deviations. Statistical analysis: Student's *t*-test.

RA animals exhibited significant decreases in skeletal muscle mass in the *EDL*, *gastrocnemius*, and *soleus* muscles, but that of *vastus lateralis* significantly increased (Figure 2).

**Figure 3:**
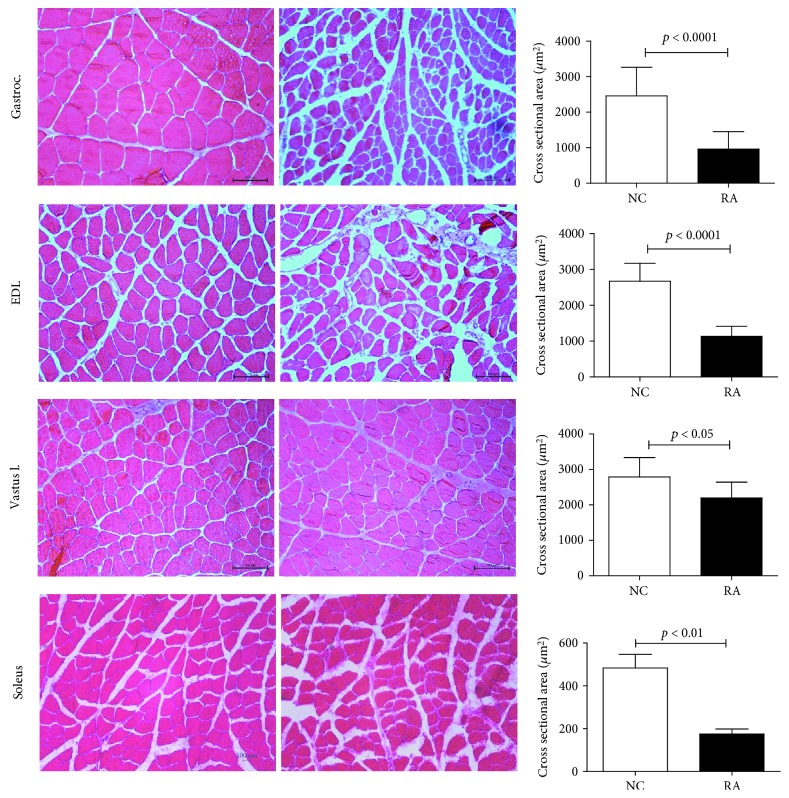
Representative H&E images and cross-sectional area of the *gastrocnemius* (a–c), *EDL* (d–f), and *vastus lateralis* (g–i) muscles depicting ultrastructural changes in female rats subjected to CIA (*n* = 10 per group) (normal control left; CIA right). 200x magnification. Scale bar represents 100 *μ*m. Statistical analysis: Student's *t*-test.

**Figure 4:**
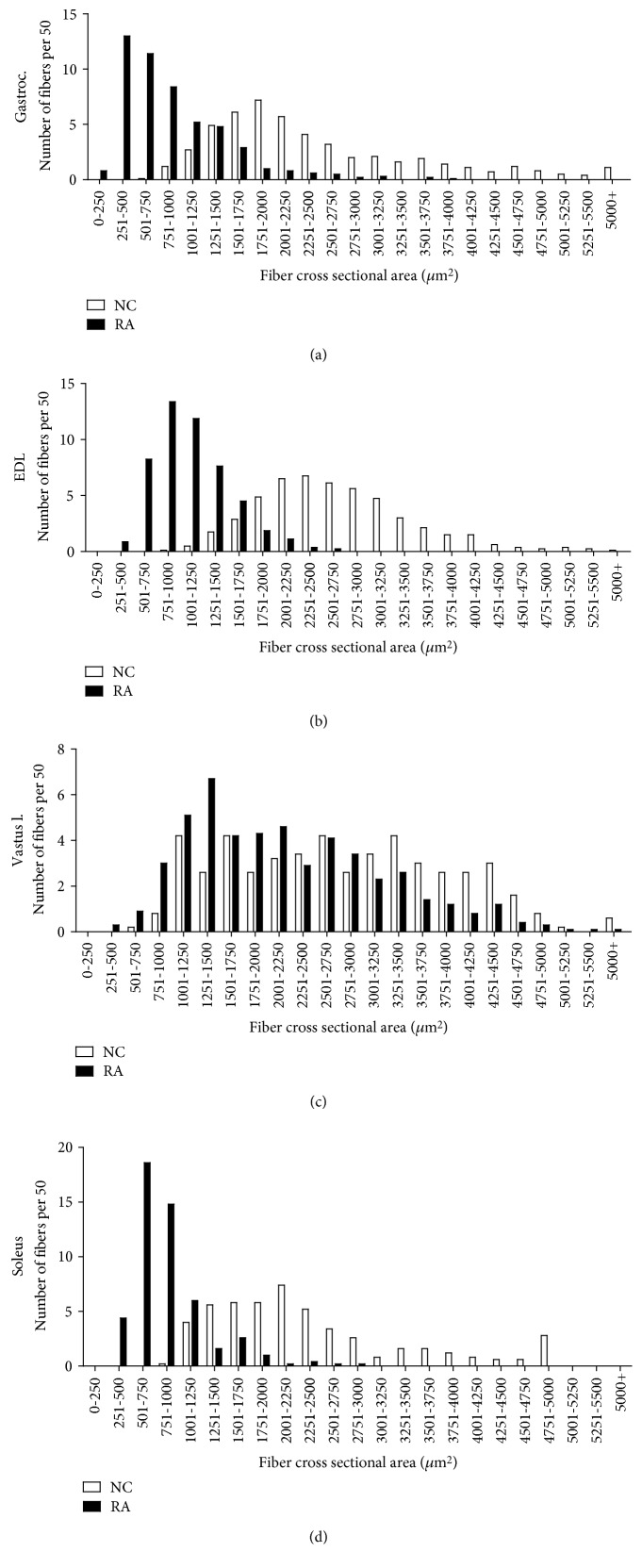
Distribution of fiber cross-sectional area across different muscle groups (*n* = 10 per group). Frequency data is expressed as number of fibers out of a total of 50 fibers counted per sample.

**Figure 5:**
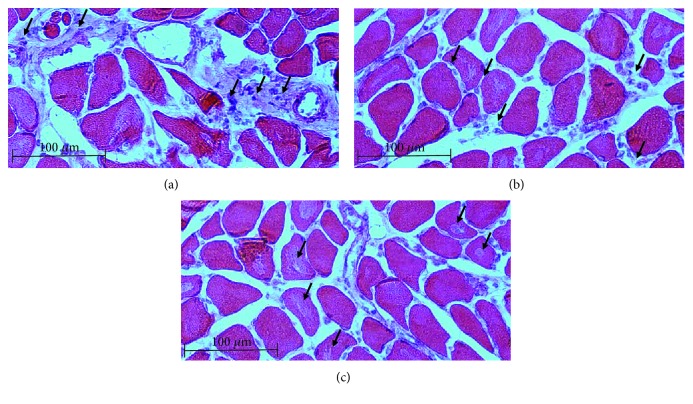
Representative high-resolution H&E stained images illustrating inflammatory cell infiltration in (a) perivascular and (b) interfiber areas, as well as (c) intrafiber necrosis. 200x magnification. Scale bar represents 100 *μ*m.

**Figure 6:**
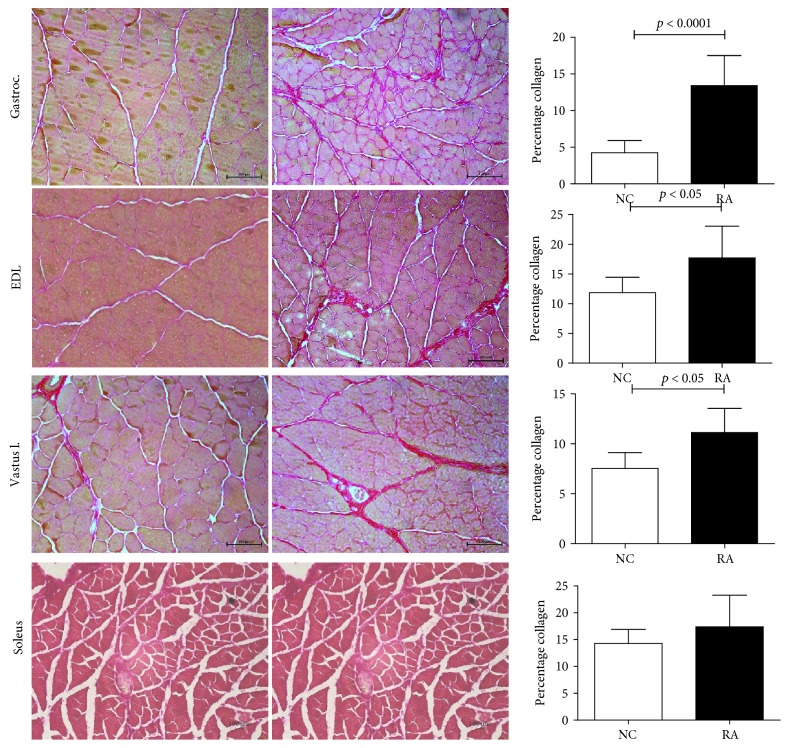
Representative images and percentage fibrosis of *gastrocnemius* (a–c), *EDL* (d–f), and *vastus lateralis* (g–i) in female rats subjected to CIA (*n* = 10 per group) using picrosirius red staining (normal control left; CIA right). 200x magnification. Scale bar represents 100 *μ*m. Statistical analysis: Student's *t*-test.

RA animals exhibited clear signs of myofiber atrophy (Figures 3 and 4), inflammation (Figure 5), and fibrosis (Figure 6) when compared to control animals. In line with the significantly decreased muscle mass, the *gastrocnemius*, *soleus*, and *EDL* muscles exhibited generalized cachexia which was characterized by a ≈60% reduction in myofiber cross-sectional area across all cells (Figures 3(a)–3(f), 3(j)–3(l)). In these muscles, a significant number of fibers undergoing degradation were visible, as well as edema and inflammatory cell infiltrate. In contrast, as suggested by the lack of muscle mass loss, *vastus lateralis* myofibers seemed least affected by RA, with cross-sectional area of fibers decreasing by only ≈20% (Figures 3(g)–3(i)). In this muscle group, the pattern of cachexia was also more varied: while some fibers of smaller cross-sectional area are visible, normal-sized fibers are still abundant. No evidence of myofiber degradation was visible in *vastus lateralis* sections analyzed.

An analysis of fiber size distribution (Figure 4) confirms these observational data. The *gastrocnemius*, *soleus*, and *EDL* muscles exhibited a shift to the left for fiber size, with very high frequency of small fiber size. The *vastus lateralis* muscle did not show a clear shift to the left, although frequency of smaller fibers did appear somewhat higher. Although inflammation was not a specific focus of this paper and specific inflammatory markers were not assessed, the RA animals clearly exhibited moderately severe levels of inflammation in the *soleus*, *gastrocnemius*, and *EDL* muscles, where inflammation was visible in the perivascular areas as well as in between individual myofibers (Figures 5(a) and 5(b)). In addition, intrafiber necrosis was visible in several cells (Figure 5(c)). These features were not clearly visible in the *vastus* muscle.

In terms of fibrosis (collagen accumulation), the *vastus lateralis* and *EDL* muscles exhibited a significant 50% increase when compared to their respective controls. A similar result was obtained in the *soleus* but did not reach statistical significance. In contrast, the *gastrocnemius* muscle was most severely compromised, exhibiting a striking 200% increase in collagen accumulation (Figure 6).

**Figure 7:**
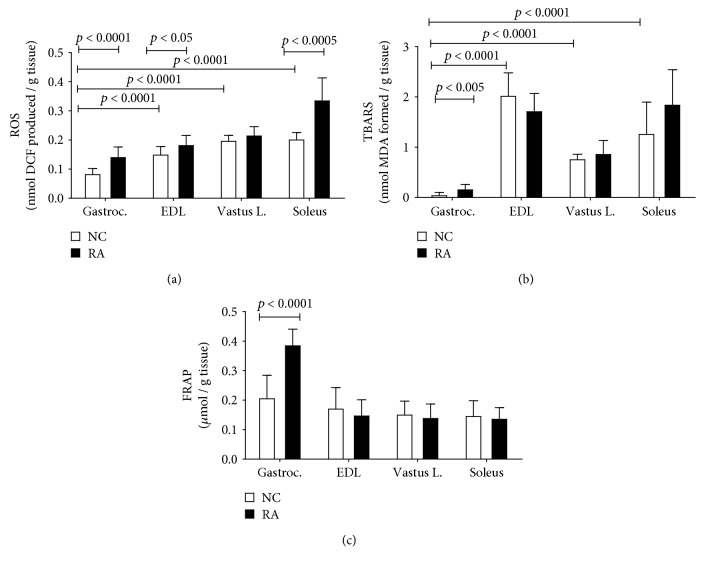
Redox status of rat muscles in female rats subjected to CIA, as measured by total ROS (a), TBARS (b), and FRAP (c) assays, respectively (*n* = 10 per group). Bars indicate means and error bars are standard deviations. Statistical analysis: 2-way ANOVA with Bonferroni post hoc tests.

In terms of redox status, total reactive oxygen species (ROS) levels seemed to correspond to muscle pathology in RA, as ROS was significantly increased in the *gastrocnemius*, *EDL*, and *soleus*, but not *vastus*, muscles of RA animals when compared to controls (Figure 7(a)). TBARS was assessed as a measure of oxidative stress-associated membrane damage through lipid peroxidation (Figure 7(b)). When considering controls only, the lowest ROS production seen in the *gastrocnemius* muscle corresponded to lowest levels of TBARS in this muscle, with highest TBARS levels measured in the *EDL* and *soleus* muscles. This relatively poorer picture in terms of oxidative damage in the control *EDL* and *soleus* muscles was not matched by a relative increase in antioxidant capacity (FRAP) (Figure 7(c)), suggesting that under normal conditions, these muscles are relatively more compromised than the *gastrocnemius* muscle in this context. In RA animals, lipid peroxidation (TBARS) levels were only significantly elevated from control levels in the *gastrocnemius* muscle (Figure 7(b)). Similarly, antioxidant capacity (FRAP) was similar and unchanged by RA in most muscles, with the exception of the *gastrocnemius* muscle, in which it was significantly increased in response to RA (Figure 7(c)).

## 4. Discussion

The current study expands on available literature by presenting a comparative assessment of different hindlimb skeletal muscles affected by CIA in a rat model. A specific novel component is the comparison of four different muscles with different fiber type distributions, including the *vastus lateralis*, in which human studies are typically conducted. Importantly, a recent study conducted in humans reported that intramuscular (*vastus lateralis*) levels of inflammatory parameters, such as the inflammatory cytokines, did not correspond to the profile in circulation [6], which highlights the importance of tissue-specific investigation and the relevance of the study reported here.

Data presented here clearly illustrates the debilitating nature of CIA and validates this model as an accurate simulation of muscle pathology in rheumatoid arthritis. Current data for the first time provides a comparative histological assessment of four muscles, three of which (the *gastrocnemius*, *soleus*, and *EDL* muscles) are directly associated with the primarily affected joint (ankle), while the other (*vastus lateralis*) is more proximal. The fact that all four muscle groups exhibited signs of cachexia, albeit it much less severe in the *vastus lateralis*, suggests that inactivity-based atrophy is not the only contributor to the pathology observed and that circulating mediators likely impact significantly on all muscle groups, irrespective of their anatomical position. This generalized muscle deterioration is in line with the human clinical profile of RA [15] and further validates our model.

The smaller fiber size observed in all muscle groups assessed in the current study could theoretically be the result of a shift in fiber type favoring type I oxidative fiber phenotype, which is generally smaller than glycolytic myofibers. This is however unlikely, since skeletal muscle wasting in COPD and other chronic diseases characterized by chronic inflammation has been reported to be associated with a shift in phenotype towards type II glycolytic fibers [16, 17]—which are incidentally also most susceptible to cachexia in general [18, 19] and specifically also to arthritis-associated cachexia [20]. This led to the hypothesis that a loss of muscle oxidative phenotype may result in increased susceptibility to inflammation [16] and oxidative stress-induced cachexia.

The inclusion of more muscle groups in the current study provided the opportunity to gain novel insights and indeed suggested that fiber type and metabolic preference alone are not major determinants of the sensitivity of myofibers to rheumatoid cachexia. In the current study, the extent of muscle fiber atrophy was similar for the *soleus*, *gastrocnemius*, and *EDL* muscles—muscles which share an anatomical site in the lower hindlimb but differ in terms of fiber type distribution and metabolic preference. The lack of more extended time points in the cross-sectional design of the current study however did not allow for assessment of change within the same muscle over time, to determine if muscles with different fiber types or metabolic preferences may respond differently over time or with disease progression. Thus, causal mechanisms potentially at play should be investigated in a study with longitudinal design including early, medium, and long-term disease progression time frames. The extent of inflammation and its effect on muscle deterioration should also be considered in such a study of longitudinal design.

In the current study, diffusely spread inflammation was clearly visible in muscle assessed, but a comprehensive assessment of inflammation was beyond the current scope. Nevertheless, skeletal muscle fibrosis has previously been linked to inflammatory processes and was quantitatively assessed. Fibrosis resulting from inflammation and associated oxidative damage in various models specifically links ROS production to modulation of transforming growth factor beta (TGF-*β*) signaling in the context of mitochondrial dysfunction [21–24]. This prompted an investigation into potential differences in redox status between the different muscles. Indeed, in the current study, even in the absence of CIA, the *vastus lateralis*, *EDL*, and *soleus* muscles exhibited higher levels of TBARS than the *gastrocnemius* muscle, which also exhibited lower ROS levels than all other muscles assessed. This is in line with previous reports of differential inherent superoxide dismutase activity, glutathione peroxidase activity, and malondialdehyde levels in different muscles (diaphragm, *soleus*, and *gastrocnemius*) [25]. When subjected to CIA, even though ROS increased in the *gastrocnemius*, *EDL*, and *soleus*, FRAP remained unchanged in the *EDL* and *soleus*, while in contrast, in the *gastrocnemius* muscle, FRAP increased significantly in response to the increased ROS of RA. Together, these data suggest that the *EDL* and *soleus* muscles may have relatively less inherent antioxidant capacity to react to oxidative stressors when compared to the *gastrocnemius* muscle, which was able to mount a significant antioxidant response. Despite this, the *gastrocnemius* muscle exhibited a worse profile in terms of malondialdehyde (MDA) production. We propose that the *EDL* and *soleus* muscles may possess antioxidant mechanisms other than those assessed by the FRAP assay, which could have upregulated activity in response to CIA and by which membrane integrity may be maintained. These mechanisms would be independent of ferric iron reduction specifically, which is assessed by the FRAP assay reported here. This interpretation is in line with the report in a model of high-fat diet-induced oxidative stress in rats, which reported that increased ROS production (reduced mitochondrial H_2_O_2_ emission, increased palmitate oxidation, and increased mRNA expression of NADPH complex) in muscle was paralleled by an increased ROS buffering capacity (increased mRNA expression of the antioxidant proteins, manganese superoxide dismutase (MnSOD), glutathione reductase, and mitochondrial thioredoxin-dependent peroxide reductase 3 and 5) [26]. Another mechanism by which this may potentially be achieved in the current context is that the *EDL* and *soleus* muscles may contain relatively higher concentrations of vitamin E, which is known to contribute to cell membrane repair after oxidative damage. In line with this, although the majority of data was generated in preclinical models and no comparison between muscles with different fiber types could be found, several beneficial effects of vitamin E were recently reported in the context of age-related sarcopenia. These included myoblast proliferation and differentiation, survival, membrane repair, mitochondrial efficiency, and maintenance of muscle mass and contractile capacity [27]. Of particular relevance to the current discussion, vitamin E supplementation was shown to prevent upregulation of muscle ring finger 1 (MuRF1) and caspase-9 and caspase-12 mRNA in unloaded rat muscle and to decrease upregulation of muscle calpain, caspase-3, and atrogin-1 (MAFbx) mRNA [28]. This would suggest that in addition to its antioxidant effect, vitamin E may also modulate atrophy via more direct inhibitory action on proteolytic pathways. The review by Chung and colleagues [27] highlighted the lack of clinically relevant information currently available on vitamin E content or protective properties in skeletal muscle, which up to now has been limited by methodological constraints. This warrants further investigations in order to elucidate how endogenous protective mechanisms may be exploited by exogenous means for therapeutic benefit.

Redox status is a major determinant of muscle pathology—and specifically chronic disease-related cachexia. The current study focused on oxidative stress resulting from reactive oxygen species. However, both oxidative and nitrosative stresses may also come into play here, as both have been implicated in cancer cachexia [29]. In addition, in rodents injected with lipopolysaccharide (LPS) to induce oxidative stress, inducible nitric oxide synthase (iNOS) was implicated as a role player in relatively greater resistance to atrophy and ubiquitin-associated protein degradation in oxidative, but not glycolytic fibers [17]. This suggests that future studies should also determine the interaction between these species but also that antioxidant treatments should be investigated as preventative modality or adjuvant treatment in RA. Indeed, astaxanthin—a powerful antioxidant—was reported to attenuate immobilization-induced increased collagen deposition via modulation of oxidative stress, most notably via altered SOD-1 and TGF-*β* signaling in rats [24]. Similarly, the antioxidant flavone mixture isoflavin-*β* was reported to attenuate toxin-induced loss of *gastrocnemius* muscle mass by preventing the oxidative modification of proteins [30].

Another observation that has remained unnoticed up to now is the fact that in the rodent model of CIA, the response in the *vastus lateralis* muscle was different from other muscles assessed here and also different from results previously reported for the *EDL* and *soleus* in rodent models of arthritis [7, 8]. Current data suggest that although the *vastus lateralis* myofibers exhibited some signs of deterioration, total muscle mass actually increased significantly. The fiber size variation in this muscle did not display the characteristic left shift associated with cachectic atrophy, although the mean cross-sectional area was somewhat reduced. This suggests that the *vastus* may have been recruited as usual for gait despite the presence of RA-related symptoms. In contrast, the severe pathology evident in the *gastrocnemius* and *EDL* muscle groups may have resulted not only from systemic effects but also due to lesser recruitment for gait and posture. This result stresses the importance of data interpretation in conjunction with clinical observations.

In terms of limitations, although not directly assessed, in our opinion, muscle mass is unlikely to have been influenced by energy intake, as animals did not exhibit clear loss of appetite. In terms of disuse as a confounding factor, although RA animals walked with a limp due to affected joints, they still moved around readily in their cages. Furthermore, a comparative study between immobilization and CIA-associated muscle atrophy has reported both proteolytic and regenerative pathways to be upregulated in RA (but not in experimentally immobilized) animals [31]. Thus, disuse is unlikely to have been a major confounding factor in the current study. Finally, edema was not quantified in the current study, but may aid in the interpretation of, e.g., the increased *vastus lateralis* muscle mass despite the presence of other signs of atrophy. Inclusion of this measure in studies investigating inflammatory mechanisms in the context of RA-associated muscle deterioration could further elucidate differences in the responses of different muscles to the challenges of RA.

## 5. Conclusion

Data presented here highlight the relevance of muscle-specific (and not just fiber-type-specific) assessment of redox profile to determine its role in muscles' atrophy response to experimentally induced rheumatoid arthritis. Furthermore, current data indicating differential responses by different muscles suggest that in human studies, it would be prudent to investigate muscle response to RA in more than one muscle. This would provide more insight into whether loadbearing and non-loadbearing muscles, or muscles at different anatomical proximities from clinically affected joints, are also differentially affected in humans in terms of disease-related cachexia. Similarly, given the inherently different redox profiles and seemingly different predominating endogenous antioxidant mechanisms at play in the different muscles as demonstrated in rats here, interventions aimed at improving redox status should take into consideration conditions in (and responses of) more muscles than just the human *vastus lateralis*, as different muscles may have different therapeutic requirements in this context.

## Data Availability

Data will be made available by authors on request.
